# Anthelmintic Efficacy of Rosemary Oil and Its Nanoemulsion Against the Monogenean Parasite, *Zeuxapta seriolae*, in Yellowtail Kingfish (*Seriola lalandi*)

**DOI:** 10.1155/anu/6414007

**Published:** 2026-02-03

**Authors:** Md Reaz Chaklader, Lindsey Woolley, Hosna Gholipour-Kanani, Masashi Maita, Gavin Partridge

**Affiliations:** ^1^ Department of Primary Industries and Regional Development, Fremantle, 6160, Western Australia, Australia; ^2^ Centre for Sustainable Aquatic Ecosystems, Harry Butler Institute, Murdoch University, Murdoch, 6150, Western Australia, Australia, murdoch.edu.au; ^3^ Laboratory of Fish Health Management, Tokyo University of Marine Science and Technology, 4-5-7 Konan, Minato, 108-8477, Tokyo, Japan, kaiyodai.ac.jp; ^4^ Oceans Institute, University of Western Australia, Crawley, 6009, Western Australia, Australia, uwa.edu.au

**Keywords:** aquaculture, cineole, nanoemulsion, pharmacokinetics, rosemary oil, *Seriola lalandi*, *Zeuxapta seriolae*

## Abstract

This study evaluated the anthelmintic efficacy of rosemary oil delivered as a nanoemulsion compared with regular rosemary oil against the monogenean parasite *Zeuxapta seriolae* in yellowtail kingfish (YTK; *Seriola lalandi*). A 20‐day feeding trial tested three dietary treatments: two levels of regular rosemary oil (providing 0.85 and 1.7 g cineole·kg^−1^ feed) and a rosemary‐oil nanoemulsion (providing 0.85 g cineole·kg^−1^ feed), against a control diet without rosemary oil. Juvenile fish (293 ± 5 g) were pre‐exposed to *Z. seriolae* oncomiracidia before the trial, resulting in an initial mean parasite abundance of 97 ± 4 per fish. At trial completion, plasma cineole concentrations were highest in fish receiving the high‐dose regular rosemary oil diet, followed by the nanoemulsion diet, and then the low‐dose regular rosemary oil. These plasma levels closely matched treatment efficacy: fish fed the high‐dose diet exhibited the lowest mean abundance of *Z. seriolae*, followed by those receiving the nanoemulsion and low‐dose diets. A similar pattern was observed for juvenile parasite recruitment. Across parasite developmental stages, all rosemary‐oil treatments significantly reduced the proportion of juvenile *Z. seriolae* relative to the control. Growth performance and feed intake did not differ among treatments. Plasma biochemistry remained unchanged, and no histopathological alterations were detected in liver or kidney tissues. Overall, these findings demonstrate that dietary rosemary oil effectively transfers cineole into the blood of YTK and reduces *Z. seriolae* infection and that application of the rosemary oil in a nanoemulsion further increases cineole uptake.

## 1. Introduction

Monogeneans are common ectoparasites infesting many farmed fish species [1] and pose a significant threat to aquaculture [2]. Chemotherapeutic treatments, such as praziquantel (PZQ), toltrazuril, mebendazole, formalin and hydrogen peroxide, have been widely used for many years to manage monogenean infections in aquaculture [3–6]. However, these approaches present several drawbacks. Bath treatments, in particular, are labour‐intensive and costly to apply at commercial scale, whereas oral drug administration is less demanding but still limited by cost, regulatory constraints and palatability issues. Furthermore, some treatments may leave residues of concern for human consumers [2, 7]. The use of herbs and herbal extracts as prophylactic or curative treatments for monogenean infections has been trialled to develop non‐chemotherapeutic parasite control strategies. In addition to having antiparasitic and immunostimulant properties [1, 8], herbal treatments are cheaper to produce than synthetic chemotherapeutics, are environmentally biodegradable, and leave no residues in the flesh which may be toxic to consumers [2, 7]. These advantages have led to several studies evaluating the potential of medicinal herbs and herbal extracts for treating monogeneans, as reviewed by [7]; but limited data is available on their effectiveness in yellowtail kingfish (YTK), *Seriola lalandi*.

YTK is one of the most important aquaculture species in Australia and supports the second fastest growing aquaculture sector in South Australia, with potential for expansion to New South Wales and Western Australia [9, 10]. Of the forty‐two parasites known to infect wild YTK throughout their biogeographic distribution [6, 11], the polyopisthocotylean monogenean *Zeuxapta seriolae* is the most significant in the YTK cage culture industry in Australia [6, 12]. Similarly, monogenean outbreaks have caused significant mortalities in *Seriola* species in sea cage farming globally, including in the Mediterranean [13], Japan [14] and New Zealand [15]. The outbreaks have affected production and caused severe economic losses. YTK in sea cages are susceptible to infection by *Z. seriolae* from wild fish; after transmission the problem is amplified by the close proximity of hosts, the parasite’s direct lifecycle and its treatment‐resistant eggs; factors which all lead to continuous reinfection. The aforementioned issues associated with currently available chemotherapeutics and the susceptibility of YTK to this parasite have prompted research efforts to investigate and develop environment‐friendly alternative therapeutic measures.

Rosemary, *Rosmarinus officinalis*, is native to the Mediterranean region and has been used broadly as a medicinal ingredient for its antiparasitic, anti‐inflammatory, hepatoprotective, antibacterial, anticancer and antioxidant properties [2, 8, 16, 17]. Rosemary contains many functional molecules [17–19], one of which is 1,8‐cineole, known to have antiparasitic properties in bath or oral treatments of cultured fish. For example, in vitro and in vivo trials against two species of monogeneans, *Neobenedenia girellae* and *Z. seriolae*, found that an ethanolic extract of rosemary in seawater and in diets was effective in controlling these monogeneans, although 100% eradication was not achieved [2]. In that study, the ethanolic rosemary extract diet contained 0.85 g·kg^−1^ of cineole and achieved a 28% reduction in parasite infestation. A more commercially relevant rosemary essential oil was also tested, but the measured cineole content of that diet was very low (0.07 g·kg^−1^) and was consequently ineffective.

Similarly, the antiparasitic effect of aqueous and ethanol rosemary extracts against the monogenean, *Dactylogyrus minutus* have been observed in in vitro and in vivo trials in common carp, *Cyprinus carpio* [8]. However, another study by Zoral et al. [20] found that oral administration of cineole at high doses can be toxic to the liver and kidney of this species.

The high volatility of essential oils and their susceptibility to decomposition when exposed to heat, humidity, light and oxygen could suppress their effectiveness in aquaculture feeds. Some of these problems can be overcome by producing nanoemulsions of the essential oils [21, 22]. The advantages of nanoemulsions of phytotherapeutics lie in superior stability, bioavailability and solubility [23, 24] which can increase therapeutic potency while reducing toxicity to the fish [25, 26]. This is due to the unique properties of nanoemulsions, such as their nanometric droplet size (10–100 nm of diameter) and large surface area which provides higher loading capacity and enhancing the solubility, and absorption of therapeutic components in intestinal epithelium [27–29]. Thus, nanoemulsions act as vehicles to increase the bioavailability of poorly soluble components in essential oils. In the latter study, a nanoemulsion of essential oil extracted from the tree species Brazilian sucupira, *Pterodon emarginatus*, demonstrated 100% anthelmintic efficacy against four different monogeneans in vivo: *Anacanthorus spathulatus*, *Notozothecium janauachensis* and *Mymarothecium boegeri* [26]. Similarly, several nanoemulsified herbal essential oils tested in another in vitro study found strong antibacterial activity against pathogenic bacteria in fish [21]. Nanoemulsions of rosemary essential oil and their addition to aquafeeds to treat parasitic infestation have not yet been studied.

The specific aims of the present study were to: (i) evaluate if cineole from rosemary oil is as effective against *Z. seriolae* in YTK as the ethanolic extract used by Ingelbrecht et al. [2]; (ii) determine if increasing dietary cineole content increases efficacy; (iii) determine if incorporating the rosemary oil as a nanoemulsion into the diet increases cineole bioavailability and subsequent efficacy and (iv) assess the hepatic and renal health of fish fed those dietary treatments.

## 2. Materials and Methods

### 2.1. Ethical Statement

All animal procedures were carried out humanly and in conjunction with the guidelines of the Australian code for the care and use of animals for scientific purposes (8^th^ edition).

### 2.2. Experimental System and *Z. seriolae* Oncomiracidia Infestation

Juvenile YTK (293 ± 5 g) were sourced from the Department of Primary Industry and Regional Development (DPIRD) marine fish hatchery in Fremantle, Western Australia and transferred to the DPIRD Annex in Geraldton, Western Australia where they were stocked into a 5000 L acclimation tank. Water flow through this tank was set at 2500 L·h^−1^ with ambient water temperature ranging from 23.3 to 27.3°C (average 25.6°C). Additional oxygen was injected into the main water distribution line to maintain dissolved oxygen at above saturation.

The fish in this acclimation tank were exposed to *Z. seriolae* oncomiracidia by placing egg strings (obtained from naturally infected YTK from a commercial sea cage) in the tank for a period of 29 days. After this period, 15 fish were sampled to determine their parasite burden prior to commencing the feed trial. Anaesthetised (Aqui‐S, 20 mg·L^−1^) fish were bathed in a 15 mg·L^−1^ PZQ solution for 10 min to remove all parasites. These fish were excluded from the feeding trial. Parasites were collected and fixed in 10% formalin and counted under 40× magnification to determine parasite loading and life stage on each fish. Infected fish were anaesthetised and radio‐frequency identification (RFID) tagged in the left shoulder, then individually weighed and randomly allocated into 12 × 5000 L tanks and tanks were randomly assigned to one of four dietary treatments (*n* = 3 tanks·treatment^−1^; *n* = 15 fish·tank^−1^).

### 2.3. Experimental Diets and Feeding

Four treatments were trialled: rosemary oil (Range Products, Welshpool WA) at a low concentration (1.24 mL·kg^−1^ feed) to achieve 0.85 g of cineole·kg^−1^ feed (based on the oil’s cineole concentration of 679 mg·mL^−1^) and thus matching the inclusion level of the ethanolic extract treatment used by Ingelbrecht et al. [2]; a high concentration (2.47 mL·kg^−1^ feed) to achieve 1.7 g cineole·kg^−1^ feed; a nanoemulsion prepared using the same rosemary oil, with the equivalent cineole concentration as the low‐dose rosemary oil treatment; and a control containing no rosemary oil. Each treatment was trialled in triplicate. Table 1 outlines the target cineole inclusion for each treatment.

**Table 1:** Cineole inclusion content and rosemary essential oil inclusion in each diet.

Dietary inclusion level	Control	Rosemary high	Rosemary low	Nanoemulsion
Rosemary essential oil inclusion (mL·kg^−1^ feed)	0	2.47	1.24	1.24
Estimated cineole^a^ inclusion (g·kg^−1^ feed)	0	1.7	0.85	0.85

^a^Based on a cineole content of rosemary essential oil (Range products, Welshpool, Australia) of 679 mg·mL^−1^ [2].

The nanoemulsion was prepared through spontaneous emulsification method by combining pre‐made oily and water phases with some modification [21]. The nanoemulsion was optimised by varying the concentrations of Tween‐80 in the aqueous phase (%, w/w) and the sonication time. The resulting nanoemulsion mixture was then homogenised in an ultrasonic bath (Bondelin Sonorex) for 10 min. Finally, the essential oil was gradually added drop by drop, resulting in a final nanoemulsion composition in the aqueous phase. The average droplet size (z‐average size) and size distribution of the obtained nanoemulsion was carried out in triplicates by photon correlation spectroscopy using a Zetasizer instrument (PCS, Nano ZEN 3000 Malvern Instruments Corporation, U.K.).

Diets were prepared using milled commercial feed pellets (3 mm, Pelagica, Ridley Agriproducts, Queensland, Australia). The control diet was prepared by adding water (450 mL·kg^−1^) and fish oil (15 mL·kg^−1^) to the ground feed. The nanoemulsion diet was prepared using the same method as the control, but 30 mL of nanoemulsion replaced 30 mL of water. The rosemary essential oil at low and high concentration, nanoemulsion, were each sonicated with 15 mL fish oil for 3 min to homogenise the oils and ensure equal distribution of the rosemary oil treatments throughout the diets. This rosemary oil/fish oil blend was added to ground commercial marine fish feed at 15 mL·kg^−1^ and mixed uniformly in a commercial food mixer for 15 min. Diets were made by adding water (450 mL·kg^−1^) to ground commercial YTK feed to form a mash, which was kneaded and cold‐pressed into 4 mm pellets using an Imperia and Monferrina Dolly pasta maker (Moncalieri, Italy). Fish were fed to satiety twice daily for 20 days and food intake was recorded daily for each tank.

### 2.4. Sampling and Analyses

After 20 days, fish were anesthetised and individually weighed. Parasite samples were collected by bathing all fish in each tank in three bathes per tank (*n* = 3–5 fish·bathe^−1^, *n* = 3 bathe·tank^−1^) using the method described earlier.

Three fish per tank were sampled to investigate potential toxicological effects of the rosemary oil. Fish were euthanised with AQUI‐S at 40 mg·L^−1^ followed by cutting of the cervical spine and whole blood was collected from the caudal vein of each fish and stored with anticoagulant in 1 mL foetal tubes (lithium heparin MiniCollect, Greiner Bio‐One, Austria). The remaining blood was spun at 3000 rpm for 10 min. The plasma was collected and stored at 20°C for biochemical analyses. Multiple parameters were then measured: liver function relevant enzymes (aspartate aminotransferase [AST], alanine aminotransferase [ALT] and glutamate dehydrogenase [GLDH]), analytes relevant to kidney function (urea and creatinine), pancreas function enzyme (lipase) and other metabolism markers (cholesterol, triglycerides and total protein). The analyses were conducted on an AU 680 Clinical Chemistry analyser (Beckman Coulter Inc., Clare, Ireland), in accordance with the methods described by Woolley et al. [30].

Liver and kidney samples were collected from each of the three sampled fish and fixed in 10% formalin for histological analyses. After fixation, tissues were dehydrated using a series of ethanol concentrations, cleared in xylene, embedded in paraffin blocks, sectioned at 5 μm slices, and stained with haematoxylin and eosin (H&E) following standard histological procedure. Histopathological assessment was observed under a light microscope (BX40F4, Olympus, Tokyo, Japan).

### 2.5. Pharmacokinetics Trial and Cineole Analysis

Since, the active component of rosemary oil, cineole, has been demonstrated to be effective at treating monogenean parasites in YTK, this trial compared the bioavailability of nanoemulsified rosemary oil against two inclusion levels of ’regular’ (non‐nanoemulsified) rosemary oil as explained earlier. The trial was also held in the DPIRD Annex in Geraldton, 9 × 5000 L in bore water flow through, with 50% exchange over a period of 4 days. The ambient bore water temperature was 22.0°C and dissolved oxygen was maintained at 100% ± 5% saturation for each of the timed trials. A total of 57 RFID‐tagged kingfish (average weight 1.57 kg) were held in a conditioning tank prior to the commencement of the trial and fed at 1% body weight per day. Diets were prepared using milled commercial feed pellets (3 mm) as explained earlier.

One fish was moved into each of the nine treatment tanks at random 1 h prior to offering treated feed, giving consistent acclimation periods for each sampling interval and three replication per diet. Each fish tag number was recorded. Fish were offered treated feed, each fish was fed to satiation for a maximum period of 30 min, with the amount of food consumed recorded. The fish were then left for the determined period either 0, 1, 2, 3, 6, 12 or 24 h post‐feeding before blood samples were taken. Different fish were used at each sample point due to regurgitation issues noted under and post‐anaesthesia with kingfish in a preliminary trial, no fish was bled twice. Approximately 2 mL of whole blood was collected as mentioned earlier using two 1 mL syringes and a 23 G needle coated with lithium heparin and transferred to 1.3 mL Lithium heparin‐coated tubes and kept chilled for a maximum period of 1‐h post sampling. Whole blood was spun in the centrifuge at 3000 g for 10 min at 4°C and plasma was collected and frozen prior to cineole concentration analysis. Mucus samples (10 mg) were collected through gentle scraping along the dorso‐lateral surface of anaesthetised fish with a blunt metal object and samples were stored at −20°C prior to freeze drying.

Plasma, mucus, and diet samples were sent to the Laboratory of Fish Health Management, Tokyo University of Marine Science and Technology, for cineole concentration analyses via gas chromatography and mass spectrometry (GC–MS) as described by Zoral et al. [8].

### 2.6. Statistical Analysis

The effect of treatment on parasite abundance and life stage prevalence, growth and feed intake was determined by one‐way ANOVA followed by a Tukey HSD test, where the tank was used as the experimental unit. Individual fish were examined to determine the effect of treatments on plasma biochemistry and parasite abundance following Ingelbrecht et al. [2]. Plasma cineole concentration (ng·mL^−1^) was subjected to area under the curve (AUC) analysis using the trapezoid rule to determine AUC_0–24 h_, *C*_max_ and *T*_max_. Variation in survival among treatments was determined by the Kaplan–Meier method with a log‐rank (Mantel–Cox) test. The significance level was set at *p*  < 0.05. The data were analysed using JMP software (version 14, SAS Institute Inc., Lane Cove, Australia). GraphPad Prism software (version 9.3, La Jolla California, USA) was used to generate all graphs.

## 3. Results

### 3.1. Dietary Cineole

**Figure 1:**
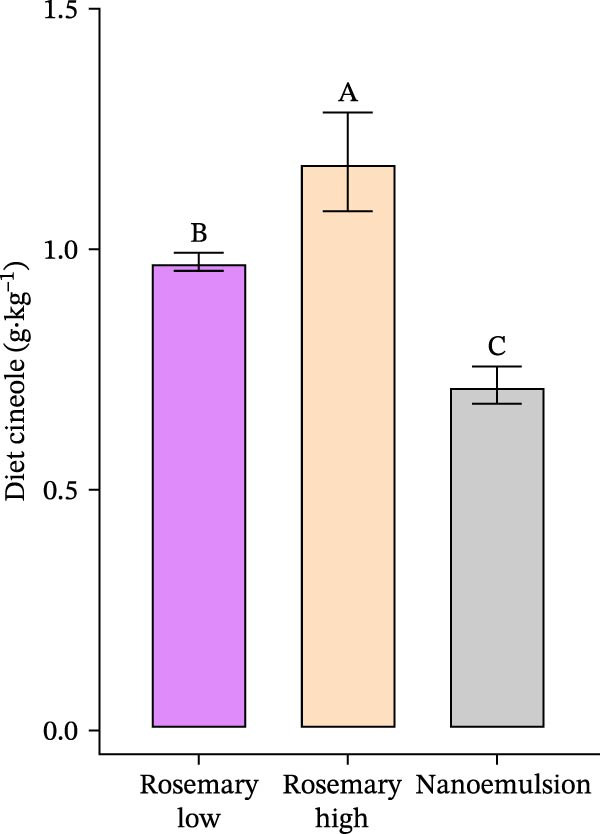
Cineole concentration (g·kg^−1^ feed) in the experimental diets, including the high and low rosemary oil treatments and the nanoemulsion treatment, demonstrated relative to their target inclusion levels. Comparisons between dietary cineole concentrations were conducted using one‐way ANOVA with Tukey’s HSD post hoc test; different uppercase letters indicate significant differences between the treatments.

Dietary analysis demonstrated that the high rosemary oil diet contained less cineole than the target inclusion of 1.70 g·kg^−1^ (achieving 1.18 ± 0.06 g·kg^−1^) (Figure 1), while the rosemary oil low contained slightly more than its target value of 0.85 g·kg^−1^ (0.97 ± 0.01). Despite these deviations, the cineole concentrations remained significantly different between the two diets (*p*<0.001). The nanoemulsion diet had the lowest cineole concentration (0.72 ± 0.02 g·kg^−1^), also below its target value of 0.85 g·kg^−1^.

### 3.2. Characterisation of the Nanoemulsion

**Figure 2:**
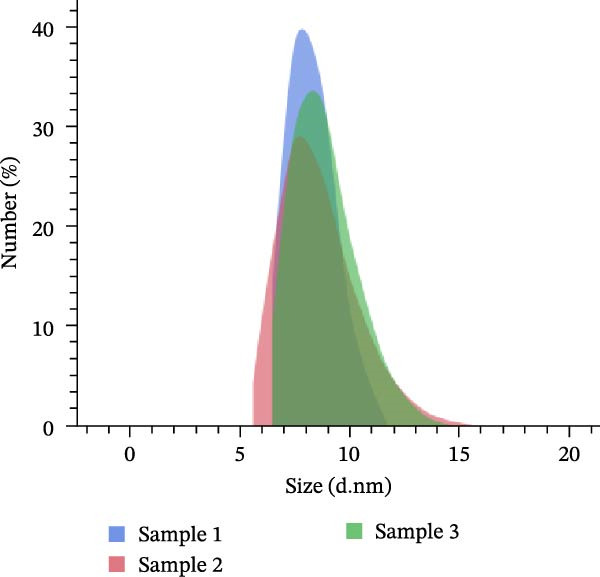
Droplet size distribution of nanoemulsion prepared from rosemary essential oil; *n* = 3.

Particle size analysis of the nanoemulsion prepared from the essential rosemary oil found the average droplet diameter to be. 9.09 ± 2.16 nm (Figure 2).

### 3.3. Parasitic Infection

**Figure 3:**
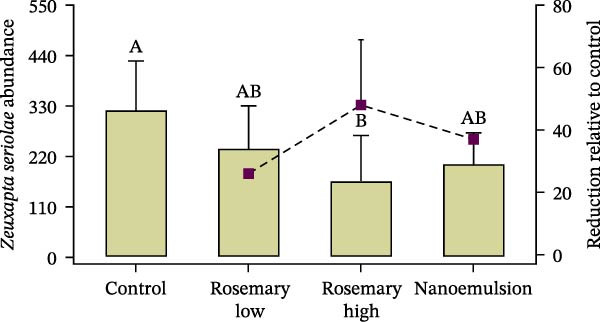
Mean abundance of *Z. seriolae* (± S.E.) infecting YTK (*n* = 9) fed either a control diet or one of three rosemary oil‐supplemented treatment diets. Percent reduction in parasite burden relative to the control diet is also presented on the secondary *y*‐axis. Treatment differences in the abundance of *Z. seriolae* were determined using one‐way ANOVA with Tukey’s HSD post hoc test; different uppercase letters above bars indicate significant differences between the treatments.

**Figure 4:**
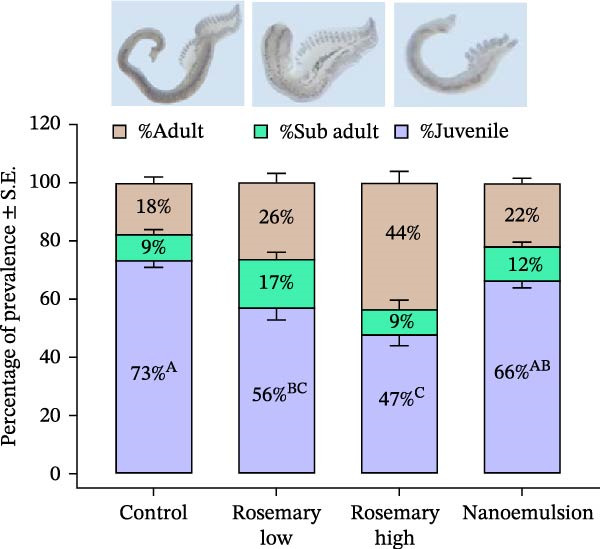
Mean prevalence (± S.E.) of adult, sub adult and juvenile *Z. seriolae* gill parasites infecting YTK (*n* = 9) following a 20‐day feeding trial. Comparisons between dietary treatments were conducted using one‐way ANOVA with Tukey’s HSD post hoc test; different uppercase letters at each prevalence value indicate significant differences between the treatments.

At the commencement of the trial, fish had a mean abundance of 97 ± 4 parasites per fish (*n* = 15; Table 2). By the end of the feeding period, parasite abundance increased substantially in the control group, reaching 322 ± 107 parasites per fish. In contrast, fish fed the rosemary oil high‐dose diet (167 ± 99 parasites per fish) and the nanoemulsion diet (204 ± 67 parasites per fish) demonstrated significantly fewer parasites abundances than the control group (*p* = 0.03; Figure 3). Relative to control, the rosemary oil‐high, nanoemulsion and rosemary oil‐low treatments reduced overall parasite burden by 48%, 37% and 26%, respectively (Figure 3). Both rosemary oil and its nanoemulsion also reduced the recruitment of juvenile *Z. seriolae*. Fish fed the high‐dose rosemary oil diet exhibited significantly fewer recruitment of juvenile *Z. seriolae* (47%) compared to the control group (70%) (*p* < 0.001, Figure 4).

**Table 2:** Mean abundance (± S.E.) and prevalence of each life stage of *Z. seriolae* recorded in YTK after 29 days of exposure to oncomiracidia.

Parasite life stage/count	Juvenile	Sub adult	Adult	Mean abundance
Number	51 ± 3	43 ± 3	6 ± 1	97 ± 4
Prevalence (%)	50	44	6	—

### 3.4. Pharmacokinetics

Plasma cineole reached peak concentrations (*T*_max_ = 1 h) in fish fed the high‐dose rosemary oil and nanoemulsion diets, with corresponding *C*_max_ values of 42.2 ± 2.6 and 30.6 ± 8.9 ng·mL^−1^, respectively. Both peaks were markedly higher than in fish fed the low‐dose rosemary oil diet (*C*_max_ = 14.4 ± 1.2 ng·mL^−1^). In addition to having a lower peak, the pharmacokinetic curve of the low‐dose diet was flatter, with a substantially longer *T*_max_ of 10 ± 2 h (Table 3).

**Table 3:** Pharmacokinetic parameters of cineole in YTK (*n* = 3) after oral administration of the three rosemary oil diets, fed to satiation for upto 30 min.

PK parameters	Rosemary low	Rosemary high	Nanoemulsion
*C*_max_ (ng·mL^−1^)	14.4 ± 1.2	42.2 ± 2.6	30.6 ± 8.9
*T*_max_ (h)	10.0 ± 2.0	1.0 ± 0.0	1.0 ± 0.0
AUC_0–24 h_ (ng·h·mL^−1^)	243.0 ± 13.9	323.7 ± 62.4	301.7 ± 9.0

*Note:* Plasma cineole concentrations were measured at 0, 1, 2, 3, 6, 12 and 24 h post‐feeding. Cineole content for each diet: rosemary oil low: 0.97 g of cineole·kg^−1^ of feed, rosemary oil high: 1.18 g of cineole·kg^−1^ of feed and nanoemulsion: 0.72 g of cineole·kg^−1^ of feed.

**Figure 5:**
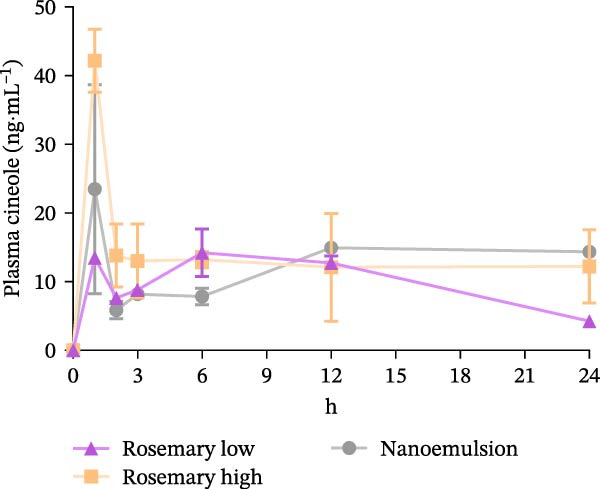
Plasma cineole concentrations in YTK over 24 h following feeding of the high rosemary oil diet (1.18 g·kg^−1^ cineole), low rosemary oil diet (0.97 g·kg^−1^) and nanoemulsion diet (0.72 g·kg^−1^). Plasma cineole were measured at 0, 1, 2, 3, 6, 12 and 24 h post‐feeding. The area under the curve (AUC_0–24_) was calculated using the trapezoidal method.

Fish fed the high‐dose rosemary oil and nanoemulsion diets also exhibited higher total cineole exposure, with AUC values of 323.7 ± 62.4 and 301.7 ± 9.0 ng·h·mL^−1^, respectively, compared with 243.0 ± 13.9 ng·h·mL^−1^ in the low‐dose treatment (Figure 5).

### 3.5. Growth, Feed Intake and Survival

At the end of the feeding trial, fish reached a mean final body weight of 646 ± 75 g, with no significant differences among dietary treatments (*p* = 0.57; Figure 6A). Feed intake did not differ significantly between treatments (*p* = 0.15; Figure 6A), and averaged 24.5 g per fish (or 3.7% BW) per day.

Figure 6(A) Final body weight (FBW) and daily feed intake (FI) (mean ± S.E.) and (B) survival of YTK fed either a control or one of the three rosemary oil supplemented treatment diets over a period of 20‐day feeding trial (*n* = 3). Differences in FBW and FI among treatments was analysed using one way ANOVA with Tukey’s HSD post hoc test. Survival was compared using the Kaplan–Meier method with log‐rank (Mantel–Cox) test.

**Figure:**
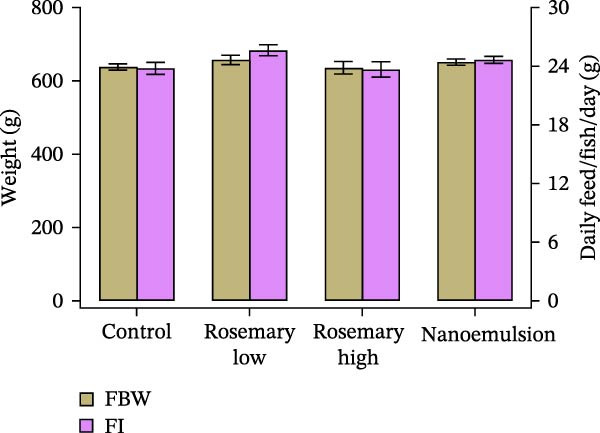
(A)

**Figure:**
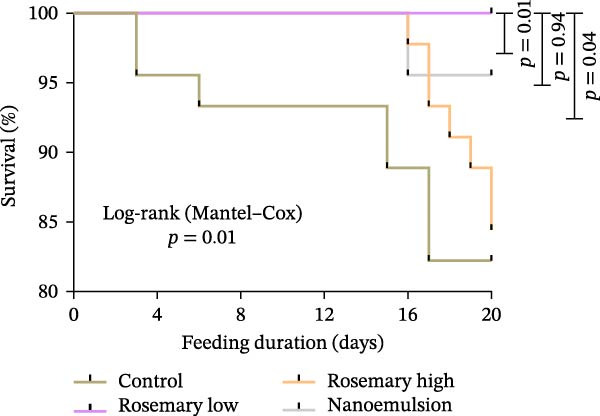
(B)

Survival varied across treatments: fish fed the low‐dose rosemary oil diet achieved 100% survival, followed by the nanoemulsion (96%), rosemary high‐dose (84%) and control diets (82%) (log‐rank *p* = 0.01, *χ* ^2^ = 11.64; Figure 6B). Survival was significantly higher in fish fed the low‐dose rosemary oil (*p* = 0.01) and nanoemulsion diets (*p* = 0.04) compared with the control diet. However, survival of fish fed the high‐dose rosemary oil diet did not differ from the control (*p* = 0.94).

### 3.6. Biochemical Responses

None of the plasma biochemical parameters measured (AST, ALT, GLDH, lipase, urea, creatinine, cholesterol, triglyceride and total protein) differed significantly among treatments (*p* > 0.05; Table 4).

**Table 4:** Plasma biochemical parameters (mean ± S.E.) of YTK fed either a control or one of three rosemary oil supplemented diets over a period of 20‐day feeding trial (*n* = 3).

Biochemical responses	Control	Rosemary low	Rosemary high	Nanoemulsion	*p*‐Value
AST	23.0 ± 2.4	35.9 ± 8.8	33.9 ± 5.7	31.3 ± 5.5	0.49
ALT	2.2 ± 0.6	2.1 ± 0.3	2.4 ± 0.2	2.9 ± 0.5	0.61
GLDH	89.7 ± 16.9	140.8 ± 32.7	131.7 ± 33.7	192.2 ± 64.3	0.30
Lipase	4.2 ± 0.4	3.9 ± 0.4	5.2 ± 0.7	4.2 ± 0.6	0.32
Urea	9.4 ± 0.7	8.5 ± 0.3	7.6 ± 0.4	8.7 ± 0.6	0.13
Creatinine	32.1 ± 3.6	27.3 ± 0.9	28.8 ± 1.5	32.8 ± 3.5	0.60
Cholesterol	6.6 ± 0.4	6.8 ± 0.2	6.6 ± 0.4	7.1 ± 0.4	0.84
Triglyceride	3.3 ± 0.4	3.0 ± 0.2	3.2 ± 0.4	3.5 ± 0.4	0.95
Total protein	39.9 ± 2.3	41.7 ± 0.8	40.4 ± 1.3	41.7 ± 1.5	0.92

*Note:* Differences among treatments were analysed using one‐way ANOVA followed by Tukey’s HSD post hoc test. Cholesterol (mmol·L^−1^), creatinine (mmol·L^−1^), lipase (U·L^−1^), total protein (g·L^−1^), triglycerides (mmol·L^−1^) and urea (µmol·L^−1^).

Abbreviations: ALT, alanine aminotransferase (U·L^−1^); AST, aspartate aminotransferase (U·L^−1^); GLDH, glutamate dehydrogenase (U·L^−1^).

### 3.7. Liver and Kidney Microstructure

**Figure 7:**
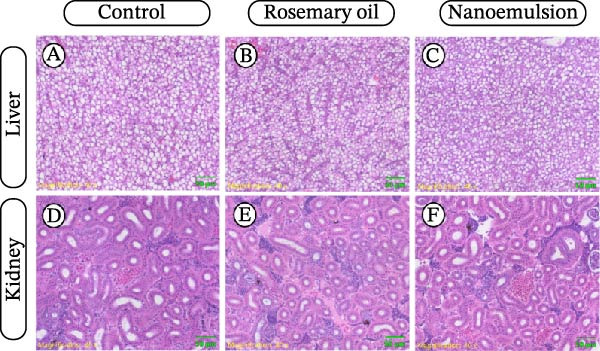
Representative micrographs of liver (A–C) and kidney (D–F) tissues (H&E stain, 40 × magnification) of YTK‐fed control and diets supplemented with rosemary oil and its nanoemulsion over a 20‐day feeding period.

Histological examination revealed no pathological alterations in the liver of fish‐fed rosemary oil or its nanoemulsion, with hepatocytes displaying normal polygonal morphology, centrally located nuclei with distinct chromatin margins and intact exocrine pancreatic tissue containing zymogen granules (Figure 7A–C). Similarly, kidney microstructure—including renal tubules, glomeruli and haematopoietic tissue—demonstrated no evidence of treatment‐related changes in any dietary group (Figure 7D–F).

## 4. Discussion

The anthelmintic effects of cineole from rosemary against *Z. seriolae* in YTK have previously been demonstrated by Ingelbrecht et al. [2], and the present study not only corroborates those findings but also provides additional refinement and insight into its application. Importantly, this work delivers the first pharmacokinetic characterisation of cineole from both rosemary oil and its nanoemulsion in YTK and links these profiles to their respective efficacies against this commercially significant parasite.

Using a dietary cineole inclusion level of 0.85 g·kg^−1^ (derived from an ethanolic extract), Ingelbrecht et al. [2] reported a 24% reduction in mature *Z. seriolae* abundance after 20 days of feeding. The first aim of this study was to determine whether rosemary essential oil could serve as an equal effective source of cineole. The comparable reduction in parasite abundance achieved here (26%) using an equivalent cineole inclusion level over the same feeding period demonstrates that cineole sourced from rosemary oil is as effective as that delivered via an ethanolic extract. Given that rosemary oil is more readily available; cost effective and easier to incorporate into commercial feed formulations, it offers a more practical alternative for industry application.

The second objective of this study was to assess whether increasing the dietary cineole inclusion from 0.85 to 1.7 g·kg^−1^ would further enhance efficacy against *Z. seriolae*. Although the intended inclusion level of 1.7 g·kg^−1^ was not reached, the high‐dose treatment achieved a dietary concentration of 1.2 g·kg^−1^, which remained substantially higher than the low‐dose diet. This elevated inclusion level resulted in markedly improved efficacy, increasing parasite removal from 26% to 48% relative to the unmedicated control. A limitation of the study was that the target cineole concentrations were not fully achieved, likely due to minor volatilisation losses during feed manufacture and natural variation in the cineole content of the rosemary oil. Nonetheless, the achieved concentrations remained clearly differentiated between treatments, enabling a valid evaluation of the dose–response relationship.

A third aim of the study was to determine if a nanoemulsion formulation could enhance efficacy relative to regular rosemary oil. The achieved dietary cineole inclusion for the nanoemulsion treatment (0.72 g·kg^−1^) was slightly below the target value of 0.85 g·kg^−1^and significantly lower than that of the low‐dose rosemary oil treatment (0.97 g·kg^−1^). Despite this lower cineole concentration, fish fed the nanoemulsion diet exhibited reduced parasite abundance (203 parasites per fish) compared with those receiving the low‐dose rosemary oil diet (235 parasites per fish) after 20 days of feeding. Whilst these differences were not statistically significant, the lower cineole content in the nanoemulsion diet indicates greater efficacy on an equivalent‐dose basis. This interpretation is further supported by pharmacokinetics analysis, which demonstrated higher cineole bioavailability in the nanoemulsion treatment (discussed in further detail in this study). Comparable findings have been reported in other species; for example, tambaqui (*Colossoma macropomum*) gill arches naturally parasitised by monogeneans (*A. spathulatus*, *N. janauachensis* and *M. boegeri*) demonstrated 100% parasite clearance following immersion in nanoemulsion containing 400–600 mg·L^−1^ of essential oil of Sucupira, *P. emarginatus*, in an in vitro trial [26]. However, to our knowledge, no studies have evaluated the efficacy of orally administered nanoemulsified essential oils against parasitic infections in marine fish, limiting direct comparisons with the present findings.

Despite significant reductions in parasite abundance across all rosemary treatments relative to the control, the absolute number of parasites continued to increase in all treatments over time, indicating that parasite recruitment exceeded removal under the conditions of the trial. A lower percentage of juvenile parasites was observed on the gills of infected fish fed rosemary‐containing diets which may reflect reduced recruitment of oncomiracidia due to fewer adult parasites, direct inhibition of oncomiracidia attachment by cineole, or a combination of these mechanisms. In a dedicated prophylactic trial, Ingelbrecht et al. [2] reported that a 30‐day feeding period with a diet containing cineole reduced—but did not fully prevent—parasite recruitment in previously uninfected fish. Consistent with those findings, neither increasing the dose of rosemary oil nor administering cineole as a nanoemulsion completely prevented recruitment in the present study.

These findings highlight that when parasite recruitment exceeds removal, overall parasite burden continues to rise, with consequential impacts on fish health and welfare. The rate of oncomiracidia recruitment is driven largely by the egg load present in the surrounding environment. Because eggs readily adhere to nets and cage infrastructure and exhibit substantial resistance to chemical treatments, reducing environmental egg burden in sea‐cage systems remains a major challenge that dietary interventions alone cannot resolve. Accordingly, unless rosemary oil dosing or delivery methods can be further optimised to achieve complete parasite removal or prevent recruitment entirely, rosemary oil is unlikely to serve as an effective stand‐alone treatment. Instead, it should be considered a valuable component within a broader integrated pest management strategy.

Feeding cineole‐containing diets for extended periods, or even continuously, represents a promising strategy to suppress or eliminate parasite recruitment. Zoral et al. [8] demonstrated this possibility in common carp (*Cyprinus carpio*) infected with the monogenean *Dactylogyrus minutus*: parasite abundance was significantly reduced after 10 days of feeding and complete elimination was achieved by day 30. However, it is unclear whether fish in that study were continuously exposed to oncomiracidia, as they were in the present trial; if not, the results cannot confirm prevention of ongoing recruitment. Additionally, *D. minutus* appears more susceptible to cineole than *Z. seriolae*, given that the cineole concentration used in the 100% efficacious diet was only 0.03 mg·kg^−1^—substantially lower than the levels required to reduce *Z. seriolae* in YTK.

As anticipated, the high inclusion of rosemary oil produced a markedly higher peak plasma cineole concentration (*C*_max_ 42.2 ± 2.6 ng·mL^−1^) compared with the low‐dose rosemary oil diet (14.4 ± 1.2 ng·mL^−1^). Fish fed the nanoemulsion diet also achieved a higher plasma cineole concentration (30.6 ± 8.9 ng·mL^−1^) than those fed the low‐dose rosemary oil diet, despite the nanoemulsion containing significantly less dietary cineole. This confirms that the nanoemulsion does offer improved bioavailability of cineole, most likely due to the substantially small‐sized droplets which increases surface area and facilitates gastrointestinal absorption [31].

The AUC data further support these observations. Fish fed the high‐dose rosemary oil diet exhibited an AUC 1.2 times greater than those fed the low dose diet (323 vs. 243 ng·h·mL^−1^), consistent with the high‐dose diet containing 1.2 times more cineole. Notably, the nanoemulsion diet produced a higher AUC than the low‐dose diet (301 vs. 243 ng·h·mL^−1^) despite containing less cineole, again demonstrating superior bioavailability. Although the PK data are presented as AUC_0–24_, plasma cineole concentrations had not returned to zero by 24 h and were highest in the nanoemulsion group at this time point. This indicates that cineole from the nanoemulsion is retained in the circulation for longer, and extended sampling would likely have shown an even greater divergence in AUC between the nanoemulsion and low‐dose treatments.

The low *T*_max_ values observed indicate rapid absorption of cineole into the bloodstream, followed by a rapid decline—similar to patterns reported in mammals, such as humans, mice and possums, following ingestion of cineole‐rich herbs or rosemary oil [32–34]. The rapid decline suggests that regular feeding of rosemary supplementations throughout the day—rather than providing a single feed—may be necessary to maintain higher plasma cineole concentrations and potentially enhance therapeutic efficacy against *Z. seriolae* in YTK .

Comparisons with other species further highlight interspecific differences in cineole pharmacokinetics. [20] reported a much higher *C*_max_ (118 ng·mL^−1^) in common carp despite feeding a diet considerably lower in cineole than used in the present study. Additionally, unlike Zoral et al. [20], no cineole was detected in the mucus of YTK. These discrepancies likely reflect species‐specific metabolic differences: YTK have a notably high metabolic rate [35, 36], which would break the cineole down more rapidly and limited its transfer into mucus layer. These findings suggest that cineole doses and feeding durations evaluated here appear inefficacious for targeting monopisthocotylean monogenean parasites, which affect YTK primarily by grazing on their mucus [37, 38].

The absence of negative effects on growth or feed intake in the present study is consistent with the findings of Ingelbrecht et al. [2] and further indicates that neither the higher rosemary oil doses nor the nanoemulsion formulation impaired performance. Average daily feed intake was 24.5 g per fish equivalent to 3.7% BW per day, which is higher than typical feeding rates reported for YTK at 23–28°C [39]. Unlike Ingelbrecht et al. [2], however, we observed no dietary influence on FCR, whereas the earlier study reported a significant reduction in feed conversion efficiency when cineole was supplied via ethanolic extract. This contrast highlights a potential advantage of rosemary essential oil over ethanolic extract as a cineole source. Our results are supported by Ebrahimi et al. [40], who supplemented beluga sturgeon (*Huso huso*) diets with up to 2% rosemary essential oil for 8 weeks without observing changes in growth or feed efficiency. Conversely, Karataş et al. [17] reported no growth benefits at 0.4 or 0.7 g·kg^−1^ rosemary powder but observed improvements in both growth and conversion efficiency at inclusion levels of 1 and 3 g·kg^−1^. Similarly, feeding upto 1% of rosemary extract and upto 3% of rosemary leaf powder improved growth and feed utilisation in common carp [16, 41]. Collectively, these findings support further exploration of higher rosemary oil doses and/or longer feeding durations, as these strategies may not only enhance parasite removal but also improve growth performance.

Although the lowest survival among rosemary treatments occurred in the high‐dose rosemary oil group, neither histopathology nor clinical chemistry revealed a cause for this mortality. Previous studies have reported no mortality in fish fed very high doses of rosemary derivatives, including beluga sturgeon fed upto 2% rosemary essential oil for 8 weeks [40] and rainbow trout fed 10 g·kg^−1^ cineole for 50 days [42]. These findings suggest that rosemary oil or cineole were unlikely contributors to mortality in the present study. Mortality is often associated with high parasite burdens, and the control group—carrying the greatest parasite loads—experienced the highest mortality. However, mortality in the high rosemary oil group, despite having the lowest parasite abundance, suggests a possible interaction between parasite burden and cineole dose, warranting further investigation.

In response to the histopathological and biochemical abnormalities reported by Zoral et al. [20] in common carp fed aqueous rosemary extract, a final aim of this study was to evaluate hepatic and renal health in YTK fed the various cineole‐containing diets. Zoral et al. [20] documented elevated plasma AST and morphological evidence of liver damage (nuclear pyknosis, cell atrophy, necrosis and irregular nuclei), as well as renal changes (pyknotic cells with vacuolated cytoplasm and tubular necrosis). The authors noted, however, that cineole levels in their study were far below toxic thresholds established in mammals, suggesting that other components of the aqueous extract may have been responsible. The absence of comparable abnormalities in the present study—despite substantially higher cineole exposure—supports this interpretation and demonstrates the safety of rosemary essential oil as a dietary additive for YTK. This lack of toxicity further justifies evaluating higher doses and/or longer‐term or continuous feeding strategies.

Beyond safety, evidence exists that cineole may enhance immune function. Taheri Mirghaed et al. [42] reported that feeding pure cineole to rainbow trout at up to 1% (10 g·kg^−1^) for 50 days improved lysozyme and complement activities, reduced cortisol levels and increased survival under crowding stress. Similar immune‐enhancing effects have been reported in the same species [17]. Such responses suggest that dietary cineole could offer broader health benefits in addition to its anthelmintic activity.

## 5. Conclusion

**Figure 8:**
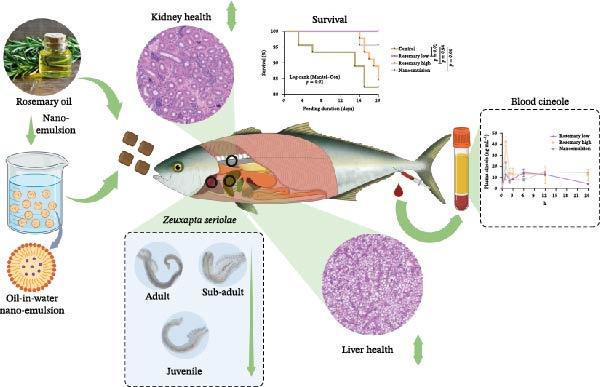
Schematic illustration summarising the effectiveness of the high rosemary oil diet (1.18 g·kg^−1^ cineole), low rosemary oil diet (0.97 g·kg^−1^) and nanoemulsion diet (0.72 g·kg^−1^) in reducing *Z. seriolae* infection in YTK.

Rosemary oil and nanoemulsified rosemary oil did not affect growth performance, feed intake or feed conversion efficiency in YTK and produced no histopathological changes in the liver or kidney (Figure 8). Plasma biochemistry, aside from elevated plasma cineole, also remained unchanged, indicating that neither higher cineole inclusion nor the nanoemulsion formulation induced hepatic or renal toxicity. Bioavailability was enhanced in the nanoemulsion diet, as demonstrated by increased *C*_max_ and AUC values. In all cases, dietary cineole was associated with reductions in the mean abundance of *Z. seriolae* and lower numbers of juvenile parasites relative to the control. However, the cineole concentrations tested were insufficient to achieve complete parasite elimination.

The absence of toxicity or growth impairment at the doses evaluated, combined with supportive evidence from other species, suggests that higher inclusion levels and/or longer‐term feeding should be explored to further improve anthelmintic efficacy. Future research should also include sensory and fillet‐quality assessments, such as sensory panels, volatile compound profiling and lipid oxidation analyses, to ensure prolonged rosemary oil supplementation does not influence consumer acceptability of YTK. Although rosemary oil is widely available as a food‐grade essential oil, its use as an antiparasitic in aquaculture is not yet commercially established; nevertheless, the favourable safety and efficacy profile demonstrated here highlights strong potential for future commercial development pending further validation and regulatory evaluation.

## Funding

This study was funded by the Fisheries Research and Development Corporation under the project “Increasing production and value of yellowtail kingfish aquaculture in warm water through improvement in feeds and disease resistance” (Grant 2017‐030).

## Conflicts of Interest

The authors declare no conflicts of interest.

## Data Availability

The data that support the findings of this study are available upon request from the corresponding author. The data are not publicly available due to privacy or ethical restrictions.
